# First insights into the movements of young-of-the-year white sharks (*Carcharodon carcharias*) in the western North Atlantic Ocean

**DOI:** 10.1038/s41598-018-29180-5

**Published:** 2018-07-17

**Authors:** Tobey H. Curtis, Gregory Metzger, Christopher Fischer, Brett McBride, Michael McCallister, Leann J. Winn, Jessica Quinlan, Matthew J. Ajemian

**Affiliations:** 1 National Marine Fisheries Service, Atlantic Highly Migratory Species Management Division, Gloucester, Massachusetts, USA; 2 Southampton High School, Southampton, New York, USA; 3 OCEARCH, Park City, Utah, USA; 4 Florida Atlantic University, Harbor Branch Oceanographic Institute, Ft. Pierce, Florida, USA; 5 New Jersey Institute of Technology, Newark, New Jersey, USA; 6 South Fork Natural History Museum, Bridgehampton, New York, USA

## Abstract

In recent years, white sharks (*Carcharodon carcharias*) have become more accessible to researchers off the northeastern U.S. as feeding aggregation sites have emerged and the population has increased. However, there has been limited research on young-of-the-year (YOY) sharks relative to older age classes in this region. Previous research indicated that YOY white sharks were most frequently observed in the New York Bight, suggesting the region serves a nursery role. To further examine the species’ use of this area, we deployed satellite and acoustic tags on ten YOY white sharks (138–166 cm total length) off Long Island, New York. The sharks remained resident in New York Bight waters through summer (August through October), further supporting the notion that the region is a nursery area. Southward movements were observed during fall, with overwintering habitat identified off North and South Carolina shelf waters. Return migrations toward the New York Bight were observed in some individuals the following spring. YOY white sharks in this heavily-populated region are exposed to anthropogenic impacts such as fisheries bycatch and coastal habitat degradation. As juvenile survival rates are important for long-term population sustainability, further research is necessary to assess the potential impacts of these activities on the western North Atlantic white shark population.

## Introduction

White sharks (*Carcharodon carcharias*) have not been as well studied in the western North Atlantic Ocean as they have in other regions of their circumglobal range^1–4^. Historically, white sharks were rarely encountered and sparsely distributed in this region^5^. However, the recovery of pinniped prey populations off the northeastern United States, and the apparent rebuilding of the white shark population in response to fisheries management^4,6^ have facilitated more reliable access to these animals in recent years. Large juvenile to adult [>2 m total length (TL)] sharks now seasonally aggregate adjacent to gray seal (*Halichoerus grypus*) haulout areas in the vicinity of Cape Cod, Massachusetts, where considerable research effort has been focused in recent years^6,7^.

Minimal research has been conducted on young-of-the-year (YOY) white sharks in the western North Atlantic as this age class has not been predictably accessible to researchers over the years^4,5^. This lack of access is due, in part, to lacking knowledge of the location and timing of parturition in this region^4,7^. However, based on a compilation of historic presence data from numerous fishery-dependent and –independent sources, Casey and Pratt^5^ first identified the New York Bight (i.e., continental shelf waters between Montauk, New York and Cape May, New Jersey) as the region with the most frequent occurrence of smaller white sharks (<2 m TL), and suggested the area could serve as a nursery area. Curtis *et al*.^4^ updated the distributional analysis of Casey and Pratt^5^ and further confirmed that YOY (<175 cm TL) white sharks most frequently occurred during summer months in waters less than 50 m deep in the New York Bight. For an area to be considered a shark nursery area, data should demonstrate that (1) YOY sharks are more frequently encountered in the area compared to other areas, (2) YOY sharks use the area repeatedly across years, and (3) YOY sharks demonstrate residency within the area for extended periods^8^. For YOY white sharks in the New York Bight, the first two criteria are supported by earlier work^4,5^, but the third criterion has not yet been fully addressed. Data on individual movements and residency patterns would help address this criterion and confirm if the New York Bight, or other areas in the western North Atlantic, serve as white shark nursery areas^8^.

Despite the long-term knowledge of YOY white shark presence in the New York Bight, to date, there have been no focused field studies on the species, and many basic questions about their distribution, movements, and habitat use in this region remain unanswered. While the large-scale movements of larger individuals (>2.4 m TL) have now been described^7^, except for a single report of a YOY white shark off North Carolina during the month of January^4^, seasonal migration patterns and overwinter habitat of YOY white sharks in this region have not been documented. Studies of YOY white shark movements are also necessary to gauge exposure to various anthropogenic impacts. White sharks are prohibited (no-retention) species in the U.S. Atlantic^9^; however, they are incidentally captured in a number of rod and reel, trawl, gillnet, longline, and trap/weir fisheries and subject to occasional bycatch mortality^4^. Due to their smaller size and nearshore distribution, YOY white sharks are comparatively susceptible to bycatch^4,10–12^. They may also be exposed to offshore energy development activities (see: https://www.boem.gov/New-York/) and coastal habitat degradation in the region. Therefore, data characterizing movement, migration, and habitat use patterns will allow managers to better assess potential impacts on this vulnerable life stage.

The purpose of this study was to describe the movements of YOY white sharks tagged in the New York Bight. This represents the first time YOY white sharks have been tracked in the North Atlantic, and provides novel insights into the spatial ecology of this life stage that complement recent work on larger white sharks^7^.

## Materials and Methods

### Capture and tagging

**Figure 1:**
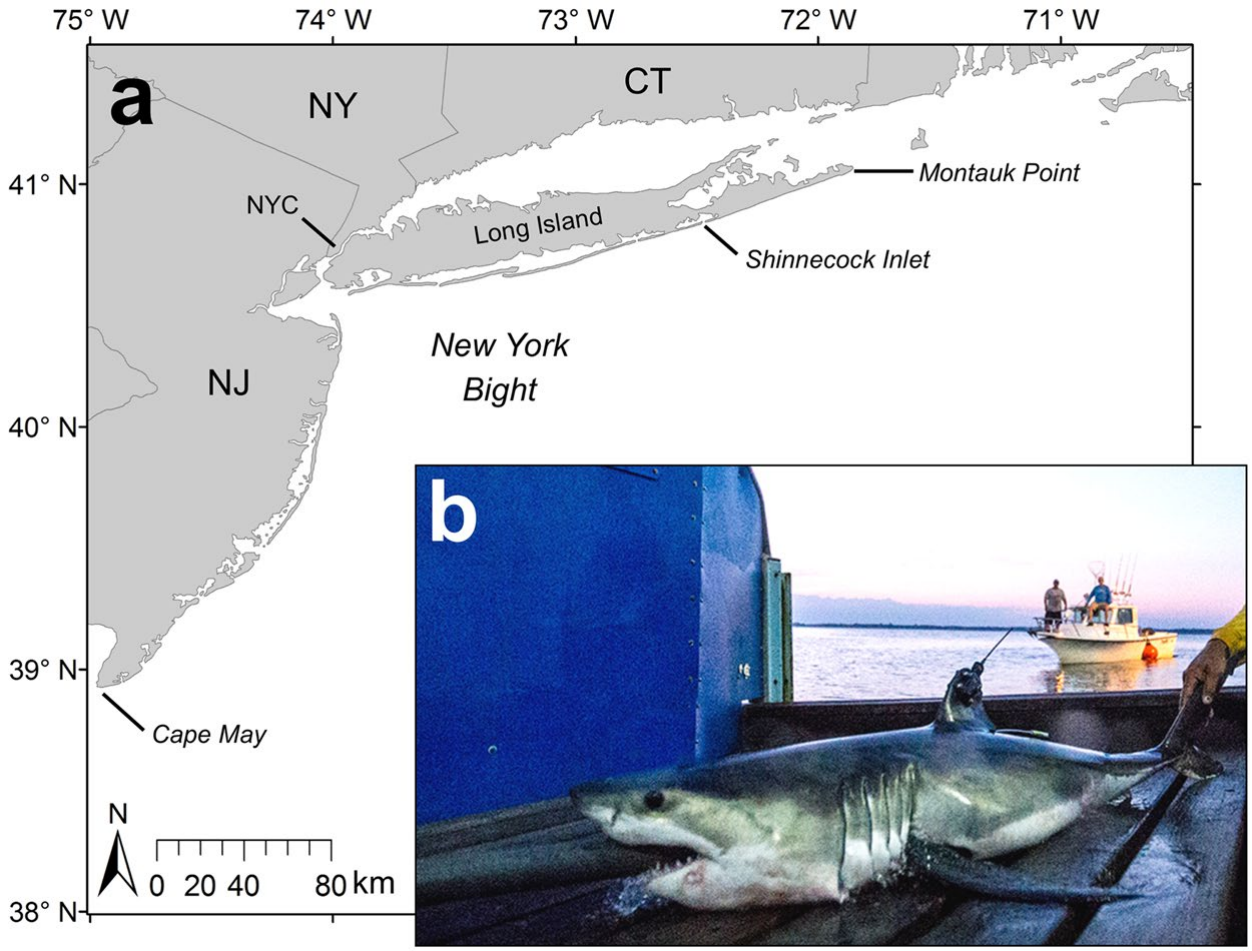
Map of the study site off Long Island, New York in the western North Atlantic (**a**) and photograph of a satellite tagged YOY white shark (WS2) on the M/V OCEARCH boat lift tagging platform (**b**). CT = Connecticut; NY = New York; NYC = New York City; NJ = New Jersey. Image copyright OCEARCH.

YOY white sharks were collected in August 2015 and 2016 by rod and reel off the south shore of Long Island, New York between Shinnecock Inlet and Montauk Point (Fig. 1a). Hooks (14/0 circle) were baited with fresh or frozen mackerel and squid and fished using standard recreational shark fishing techniques, except for one individual (WS2) that was caught on a 12/0 J-hook while actively jigging in a school of baitfish. Fight times once hooked ranged from 4–16 min. Each shark was subsequently guided to a boatlift platform on the M/V OCEARCH where it was raised out of the water for sampling and tagging (Fig. 1b). The head and eyes were covered with a wet towel, hooks were removed, and raw seawater was pumped over the gills via a hose inserted in the mouth.

All sharks were sexed and measured over the curvature of the body to the nearest cm precaudal length, fork length (FL), and TL. Sharks were fitted with various tag types (different sharks received different combinations of tag types), including satellite-linked Smart Position or Temperature Transmitters (SPOT-258A, tag life up to 3 years, Wildlife Computers, Redmond, Washington), individually coded acoustic transmitters (V16-6H, tag life 10 years; VEMCO, Bedford, Nova Scotia) to be monitored by the Atlantic Cooperative Telemetry (ACT) network, pop-up satellite archival tags (PSAT; SeaTag-MOD, Desert Star Systems, Marina, California), and conventional dart-style M-tags from the National Marine Fisheries Service (NMFS) Cooperative Shark Tagging Program. SPOT tags were attached by nylon bolts and rotated to match the leading edge of the first dorsal fin, allowing the antenna to transmit to the Argos satellite system whenever the shark’s fin was above the ocean’s surface. The PSAT and M-tags were externally attached via a stainless steel or plastic umbrella-style dart and tether into the dorsal musculature near the base of the first dorsal fin. Acoustic transmitters were surgically implanted into the coelomic cavity via a 4-cm incision and stitched closed with Prolene monofilament sutures. All sampling and tagging procedures were completed in 10–19 min, after which the boatlift was lowered back into the water for release. These handling and tagging methods were carried out in accordance with all applicable guidelines and regulations under permits issued by NMFS and the New York State Department of Environmental Conservation.

### Data analysis

Argos positions (location classes 0, 1, 2, and 3) received from SPOT-tagged sharks were chronologically integrated with passive acoustic detections received from the ACT network to produce a time-series of position estimates for each shark. The spatial accuracy of the position estimates varies with the detection type, from approximately 0.4 km for acoustic and Argos location class 3 positions, to over 5 km for Argos location class 0 positions^13^. Erroneous positions occurring on land, or a result of unrealistic rates of movement (>10 km h^−1^), were removed^14^. In general, SPOT position quality and frequency declined over time from release, with 81% of total Argos positions received in the first 3 months after release. It is not known why SPOT position quality and frequency declined with time, but it may be due to bio-fouling of the transmitter, tag malfunction, sharks spending less time at the surface, damage to the sharks’ dorsal fins, or a combination of these factors. Thus, track segments from the release dates in August through October 2016 were regularized to single daily positions using the Resample tool in the Movement Ecology Tools for ArcGIS ® (ArcMET) toolbox (10.2.2 v3) to provide standardized track steps for movement analyses. The tool permits systematic downsampling of oversampled temporal windows within a movement data set, and interpolates new positions at specified intervals (e.g., 24 h)^15^. The downsampled daily positions from August through October were used to generate a cumulative fixed kernel utilization distribution^16^ of the sharks during this period, with results summarized using 25%, 50%, 75%, and 95% volume contours (25% and 50% contours delineating core areas of use)^11^. The final utilization distributions were clipped to exclude areas not occupied by the tracked sharks (e.g., land, inshore waters).

We characterized habitat use with respect to bathymetry, sea surface temperature, and distance from shore. Bottom depth from the ETOPO1 Global Relief Model^17^ (1 arc-minute resolution) was matched to each raw position in ArcGIS (v. 10.3, ESRI, Redlands, California). Remotely-sensed daily sea surface temperatures (Global High-Resolution Sea Surface Temperature Pilot Project^18^, 1 km resolution, NOAA CoastWatch) were matched to each raw position based on its latitude, longitude, and date. Because the transmitters used in this study did not archive or transmit water temperature observations, matching satellite sea surface temperature data to positions was the best available method to characterize temperature distributions of the sharks^14^. Distance to shore for each raw track position was also calculated using ArcGIS.

To describe long-term (November 2016 onward) movements, only the raw Argos and acoustic positions were analyzed due to the irregular and large intervals between detections. However, the tracks of multiple individuals were sufficient to preliminarily characterize the post-summer movements and migration of YOY white sharks. We used a linearity index as a metric of resident versus transient tracks in specific areas and times^19^. The linearity of a track segment was calculated by dividing the straight-line distance between the first and last positions by the total track distance for the defined period, resulting in values between 0 (completely nonlinear movements indicative of a resident state) and 1 (completely linear movements indicative of a transient state).

### Data availability

Argos satellite tag positions used in the current study are archived and publicly viewable on the OCEARCH Global Shark Tracker website: http://www.ocearch.org/. The complete tracking and environmental datasets analyzed during the current study are available from the corresponding author on reasonable request.

## Results

A single YOY white shark (WS1; 147 cm TL) was captured near Shinnecock Inlet in August 2015 and tagged with only a PSAT and M-tag. The PSAT from WS1 detached prematurely after 45 days, and only the pop up location was received (Table 1). Thus, only the pop up location from this individual was used in subsequent analyses. An additional nine individuals (138–166 cm TL) were captured near Montauk Point in August 2016 and each was tagged with a SPOT, acoustic, and M-tag (Table 1). Of these nine white sharks, movement data were received from five males (WS3, WS4, WS5, WS7, WS10) and three females (WS2, WS8, WS9). Only a single Argos position was received from WS6 after a period of six days post-release and it was not detected on the ACT network (possibly due to tag malfunction, the behavior of the shark preventing detection – e.g., rarely surfacing for SPOT transmission and moving offshore away from ACT coverage, or delayed mortality due to capture stress or predation). Therefore, this individual was not included in subsequent analyses. Following release, the remaining eight sharks were tracked for periods of 20–305 days (median = 111 days). During the April through October period, the overall mean (±1 SD) rate-of-movement for the YOY white sharks was 0.70 ± 0.83 km·h^−1^, with a maximum of 6.03 km·h^−1^. Collectively, across the complete track durations, these sharks were tracked in SSTs of 10.5–25.0 °C and over bottom depths of 5–286 m.

**Table 1:** Summary of information for white sharks tagged off Long Island, New York in 2015 and 2016.

Shark ID	Sex	TL (cm)	Date Tagged	Track Duration (d)	Track Distance (km)	Argos Positions	Acoustic Positions
WS1	F	147	25-Aug-2015	45	398	1*	n/a
WS2	F	142	19-Aug-2016	273	1658	43	16
WS3	M	158	20-Aug-2016	151	1340	39	8
WS4	M	138	21-Aug-2016	111	549	30	7
WS5	M	166	21-Aug-2016	81	199	7	2
WS6	M	148	21-Aug-2016	6	n/a	1	0
WS7	M	158	22-Aug-2016	20	396	14	5
WS8	F	155	23-Aug-2016	48	80	3	4
WS9	F	162	23-Aug-2016	284	338	24	2
WS10	M	162	23-Aug-2016	305	915	88	25

The Argos and acoustic positions represent counts of unique locations or receivers contributing to the final tracks (excluding interpolated positions).

*PSAT pop-up location only.

### Summer-fall movements

**Figure 2:**
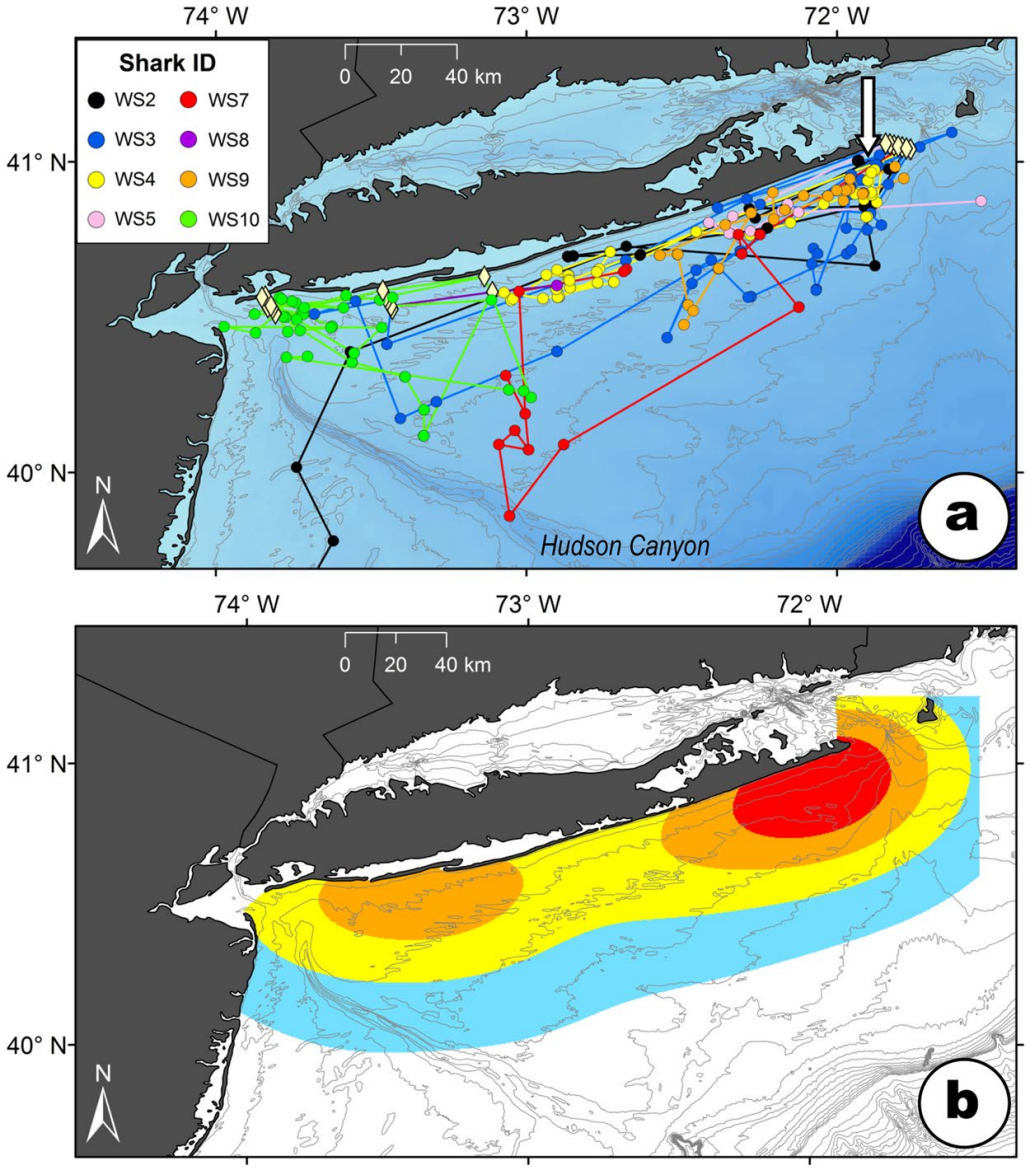
Tracks of eight YOY white sharks (**a**) and kernel utilization distributions (blue = 95%; yellow = 75%; orange = 50%; red = 25%) of the tagged sharks (**b**) off Long Island, New York, during August through October, 2016. The arrow indicates the tagging location. Diamond symbols represent locations of acoustic receivers where YOY white sharks were detected. Bathymetric contours (gray lines) are in 10 m increments.

Following release, all YOY white sharks remained off Long Island for the duration of the summer and into the fall (August through October) (Fig. 2a). During this period, the linearity indices for all individuals were very low (<0.01), indicating highly nonlinear movement paths suggestive of residency. While positions spanned coastal waters off the entire 197 km length of Long Island (Fig. 2a), the highest density of positions (i.e., 25% and 50% utilization distributions) occurred between Montauk Point and Shinnecock Inlet suggesting the region was a focal area of activity for multiple sharks (Fig. 2b). While the sharks ranged from the surf zone to 90 km offshore, over 97% of locations during August through October were within 20 km of Long Island’s south shore (Fig. 2a). The sharks generally travelled parallel to the shoreline, over depths of 5–75 m (mean = 17 ± 7 m) (Fig. 2a). The sea surface temperatures experienced by the sharks during this period were 16.2–25.0 °C (mean = 21.4 ± 1.6 °C).

### Seasonal migration and overwinter habitat

**Figure 3:**
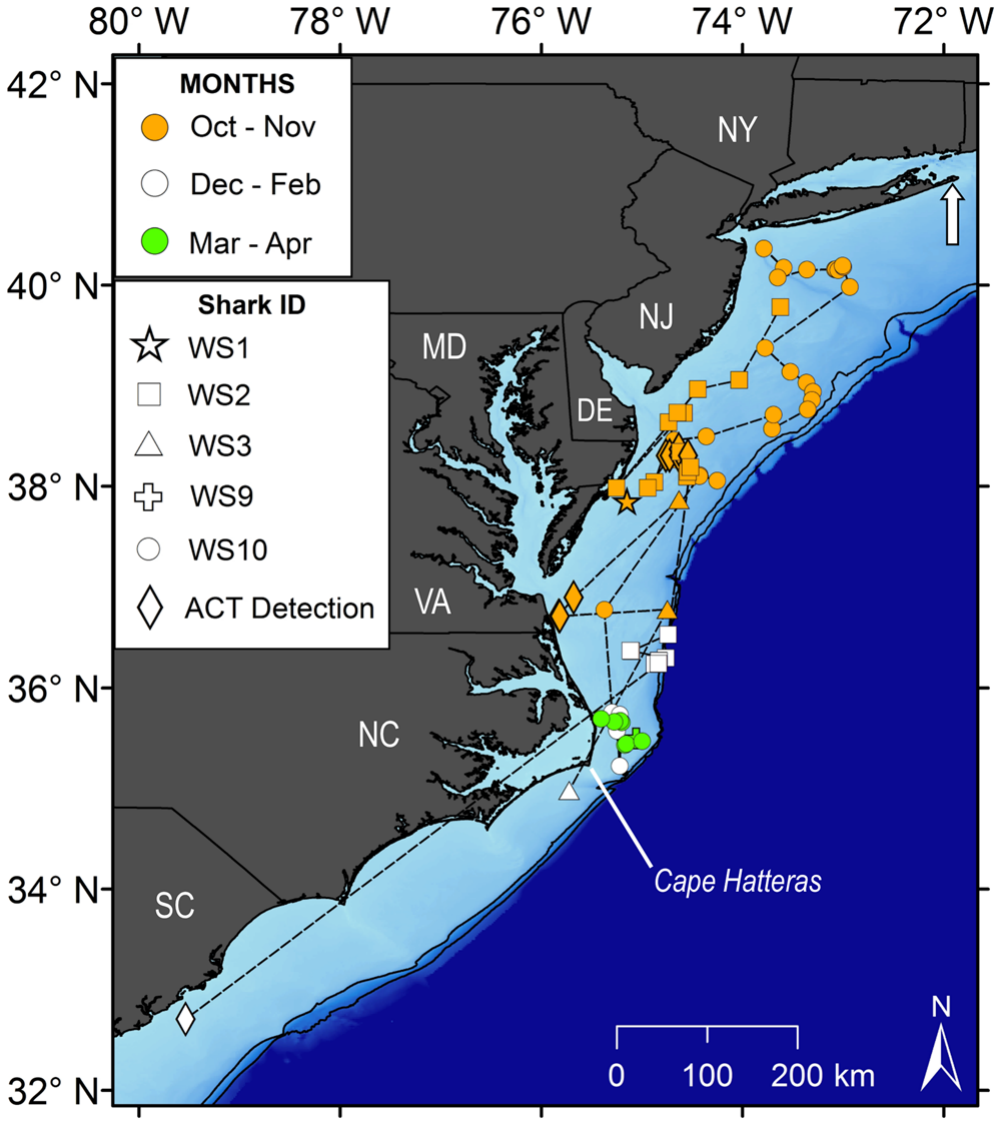
Late-fall and overwinter (October through April) tracking positions of five YOY white sharks tagged off Long Island, New York. The arrow indicates the August 2016 tagging location. NY = New York; NJ = New Jersey; DE = Delaware; MD = Maryland; VA = Virginia; NC = North Carolina; SC = South Carolina. Solid black lines are the 100 and 200 m bathymetric contours denoting the edge of the continental shelf.

Post-summer (November onward) movement observations were available from five white sharks (WS1, WS2, WS3, WS8, and WS9). These individuals departed the New York Bight during October and November, and moved southward over continental shelf waters (Fig. 3). Satellite and acoustic detections were less frequent during late fall, but there appeared to be only limited residency (linearity >0.9) in the mid-Atlantic Bight between New Jersey and Virginia (Fig. 3). By December, all sharks were located off North Carolina, with linear displacements 550–720 km from the release location. The southernmost detection was from WS2 near Charleston, South Carolina on 29 January 2017, approximately 1160 km from its release location (Fig. 3). From December 2016 through April 2017, sharks appeared to remain resident (linearity <0.1) off the Carolinas, with a focal area off the Outer Banks north of Cape Hatteras, (Fig. 3). During this period, the tagged sharks occurred over bottom depths of 9–286 m, but excluding two outlier positions beyond the edge of the continental shelf, the mean depth was 33 ± 13 m. The sea surface temperatures experienced by the sharks during this period were cooler than during summer/fall: 10.5–23.5 °C (mean = 16.6 ± 1.6 °C).

**Figure 4:**
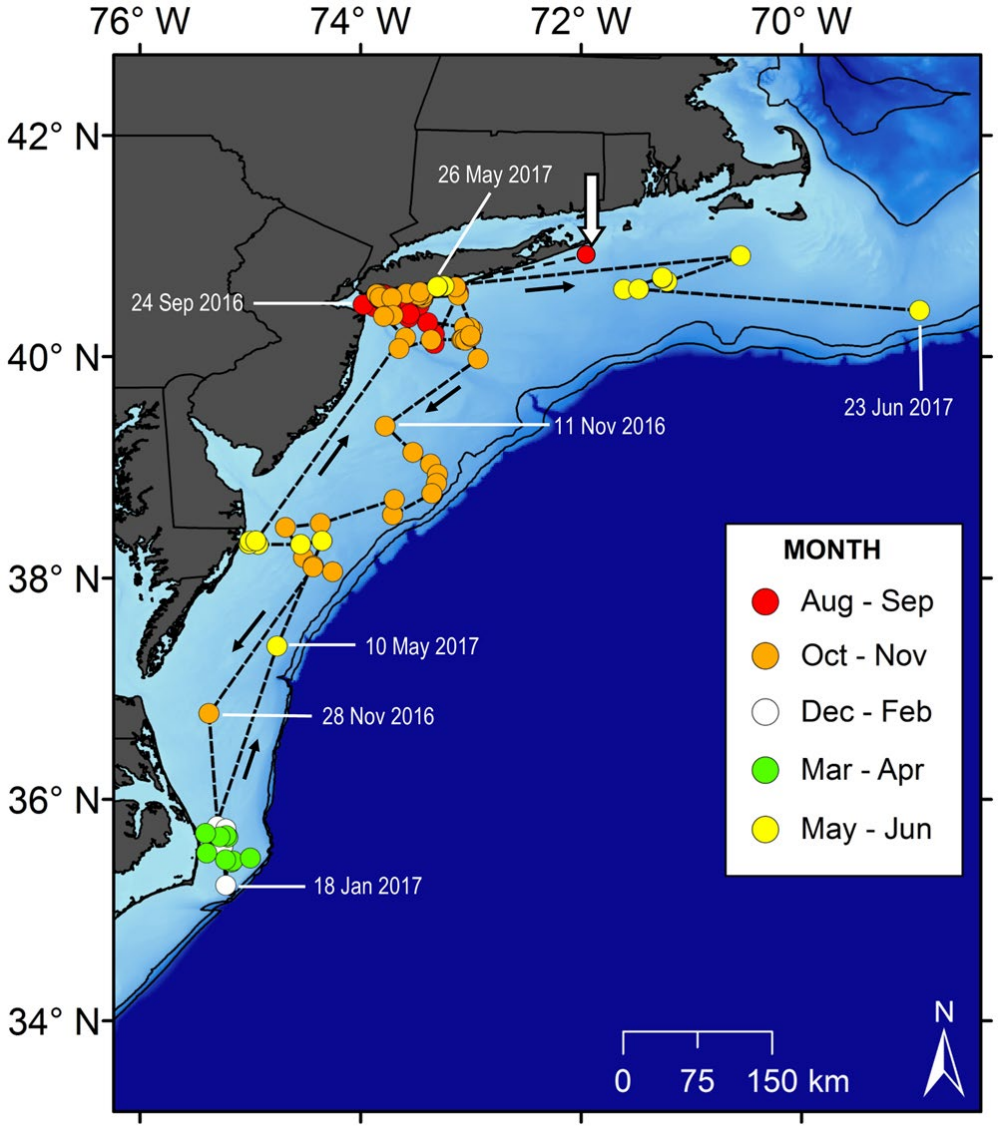
Complete 305-day track of a single YOY white shark (WS9), August 2016 to June 2017. The white arrow indicates the tagging location (August 23, 2016). Black arrows denote the directions of movement. Solid black lines are the 100 and 200 m bathymetric contours denoting the edge of the continental shelf.

Three individuals (WS2, WS8, and WS9) were detected moving back northward along the coast in May and June 2017. Only one tagged shark (WS9) provided enough observations to describe a full annual loop migration (Fig. 4). This individual was tagged on 23 August 2016, and remained resident off the south shore of Long Island until mid-November when it began moving south. During the period 6–23 November 2016, WS9 moved from the New York Bight waters north of Hudson Canyon southward to coastal waters off Virginia; a minimum track distance of 334 km in 17 days (19.6 km d^−1^). The shark remained off North Carolina from December 2016 through April 2017, and began its return migration northward in May 2017 (Fig. 4). From 20–26 May 2017, WS9 migrated northward from off the eastern shore of Maryland back to the nearshore waters off Long Island; covering a minimum distance of 377 km in 5.5 days (69.1 km d^−1^) (Fig. 4). Subsequently, during June 2017, WS9 traveled east of Long Island over continental shelf waters south of Massachusetts, as far as the edge of Georges Bank where its track ended.

## Discussion

The tracking data from the present study provide the first descriptions of the movements and seasonal migrations of YOY white sharks in the North Atlantic Ocean. Integrated SPOT and acoustic tag positions complemented one another and provided comprehensive information on movements. In the initial months post-release, SPOTs generally provided more positions and movement data compared to the acoustic data. However, as SPOT transmissions became less frequent with time, gaps in some satellite tracks were complemented with acoustic detections. As the battery life of the acoustic tags is up to 10 years, with sustained acoustic monitoring infrastructure along the U.S. east coast there is the potential to observe changes in white shark distribution, habitat use, and migration over ontogeny from YOY to large juvenile age classes. The continued use of multiple electronic tag technologies, with complementary capacities for data collection, will create the most complete animal tracks and best characterize long-term movement patterns.

The tracking data from this study revealed that the summer/fall (August through October) distribution of YOY white sharks was generally limited to the New York Bight, with focal areas along the southeastern shores of Long Island. This pattern of residency spanning multiple months, in addition to the previously documented higher frequency of occurrence of YOY white sharks in this area across many years^4,5^, confirms that the New York Bight functions as a nursery area under the Heupel *et al*.^8^ criteria. Nursery areas are generally considered to maximize individual fitness and survival rates of juvenile sharks through reduced risk of predation and high prey availability, thereby disproportionately contributing to recruitment compared to other areas^8,20^. Potential predators (e.g., larger sharks), are uncommon in the shallow nearshore waters of the New York Bight, providing YOY white sharks a refuge from potential predation pressure. The most likely predator of YOY white sharks in this region would be larger conspecifics; however, large white sharks do not appear to frequent the nearshore areas where YOY white sharks spend most of their time^4,7^. The region is also known for its high overall productivity and prey diversity, including high abundances of demersal and pelagic fishes and invertebrates^5,21^. It is not yet known if pupping occurs in or near the New York Bight, but it is apparent that pups enter this region in the months following parturition and use the area through their first summer of life.

YOY white sharks moved out of the New York Bight in the late fall. Directed movements southward along the coastline, with little evidence of residency in the mid-Atlantic region, indicate this portion of the continental shelf may be a migratory corridor. This pattern is consistent with the observed seasonal distribution shift of YOY and juvenile white sharks in eastern Australia^11,22^ and southern California^10,12^, and also larger juvenile, subadult, and adult white sharks in the western North Atlantic^4,5,7^. Larger white sharks in the western North Atlantic more frequently overwintered south of Cape Hatteras or in offshore pelagic waters^4,7^. However, the YOY white sharks tracked in this study spent their winter months mostly north of Cape Hatteras. More data are needed to clarify YOY white shark migration patterns, but it is likely that these smaller individuals migrate shorter distances than larger individuals; a pattern observed in a variety of species^23^. This pattern appears to maintain a size-based spatial segregation within the white shark population throughout much of the year. Additional research is needed to provide more information on the ecology and behavior of YOY white sharks in their overwintering habitat, now that it has been identified. The observed return migration of at least one individual to the New York Bight, and the documented presence of juveniles (age 1+) in this area^4,5^, suggests that white sharks may demonstrate site fidelity to this nursery area in early life. However, multi-year observations from additional individuals are needed to confirm this pattern.

The movements of the sharks tracked in this study generally conform to patterns observed in YOY and juvenile white sharks tracked in other regions, including southern California^10,12,24,25^, and eastern Australia^11,22^. Young white sharks in all of the regions examined generally appear to be coastally-oriented, spending the majority of their time over insular continental shelf waters, in temperate sea surface temperatures of 10–25 °C. Shifts in coastal latitude occur seasonally, largely in response to temperature changes. White shark nursery areas, therefore, generally appear to have globally consistent habitat characteristics that are likely influenced by their thermal preferences and prey availability. As individual white sharks in the western North Atlantic grow, their distribution and migrations appear to expand^4,7^. Additional research is necessary to more specifically quantify the ontogenetic shifts in spatial ecology of this important marine apex predator.

Continued electronic tagging research is needed to more fully characterize YOY white shark movements, migrations, and habitat use in the western North Atlantic. In particular, using tags that measure vertical activity in the water column (e.g., archival, accelerometer, or video tags) would provide valuable data on depth and temperature preferences and potential foraging activity in the nursery area. Very little is known of YOY white shark diet composition, with the available stomach contents data from this region suggesting a prevalence of demersal and pelagic bony fishes and smaller elasmobranchs^5^. Non-lethal sampling methods including stomach eversion, stable isotope analysis, genetic techniques, and animal-borne imaging technology may improve our understanding of their foraging ecology and trophic role in the New York Bight ecosystem.

Fisheries and ocean resource managers can use information from this study to better assess the impacts of human activities on these YOY white sharks and their habitats. Juvenile and YOY white sharks in the New York Bight occur as bycatch in recreational rod and reel fisheries, as well as in commercial trawl, gillnet, longline, and trap/weir fisheries^4^. Estimates of total discards and post-release mortality rates in these gears remain unknown, and should be a priority for future white shark research. However, given evidence that white shark relative abundance in the western North Atlantic is increasing over recent decades^4^, fishing mortality rates may currently be sustainable. While considered less of a potential threat than overfishing, exposure to coastal habitat degradation and possible habitat modification from ocean energy development activities may also be assessed with this new information. NMFS already includes the waters of the New York Bight within its “essential fish habitat” designations for YOY white sharks^26^ and the movement data from the present study may help further refine that designation and its utility in habitat conservation. Collectively, new white shark movement data from this study, and recently published work^7^, have expanded the body of information available to inform conservation efforts in the western North Atlantic white shark population.
